# P251: Prospective survey of the practice of curative antibiotic treatment in an infectious diseases department in Dakar

**DOI:** 10.1186/2047-2994-2-S1-P251

**Published:** 2013-06-20

**Authors:** NM Dia, R Ka, YZ Ismail, NM Manga, SA Diop, L Fortes, NA Lakhe, D Ka, V Ciss, AI Sow, M Seydi

**Affiliations:** 1 Infectious Diseases Department, Dakar, Senegal; 2 Laboratory of Bacteriology, FANN Teaching Hospital, Dakar, Senegal

## Introduction

The good use of antibiotics stays the best means to protect available ones. Our work aims to estimate the practices of prescription of antibiotics according to the national recommendations in a reference department.

## Methods

A descriptive and analytical prospective audit was conducted from April 2nd till July 2nd, 2012 from all cases of hospitalized patients having benefited from an antibiotic treatment with curative aim.

## Results

The study concerned 170 patients with a total of 267 prescriptions of antibiotics. The average age was 41 years [15-86 years] and the sex ratio 1.5. The majority of the prescriptions were issued by -doctors in specialization (61.8 %) and- students in the last year of medicine (37.6 %). The main listed infections were: pneumonia (28.3 %), meningitis (21.80 %), opportunist infections during HIV infection (14.70 %) and tetanus (13.52 %). From the point of view of the prescription, 57.70 % of the patients had received a monotherapy: essentially ß-lactames (55.43 %), imidazoles (16.5 %) and macrolides (13.9 %). The most commonly used antibiotic combinations were: Amoxicillin-acid clavulanique-spiramycin (37.66 %), ceftriaxone-metronidazole (11.7 %), ceftriaxone-metronidazole-gentamicin (10.4 %). The rate of conformity of the antibiotic prescription with the national recommendations was 74.7 % in particular for the posology (92.50 %), the frequency of administration (95.88 %), the duration of treatment (55.43 %). Only 15.9 % of antibiotic treatments were re-evaluated at the end of 48 hours with a modification of the antibiotic regimen in 60 patients (35.5 %), in particular a broadening of spectrum (83.33 %). The average length of stay was of 14±11 days and mortality was 69.18 %.

## Conclusion

The rational use of antibiotics is essential to protect available drugs. It would be necessary to train the physicians and to advertise the user guide of antibiotics to improve the quality of the prescription.

## Competing interests

None declared

